# Urinary and Dietary Analysis of 18,470 Bangladeshis Reveal a Correlation of Rice Consumption with Arsenic Exposure and Toxicity

**DOI:** 10.1371/journal.pone.0080691

**Published:** 2013-11-15

**Authors:** Stephanie Melkonian, Maria Argos, Megan N. Hall, Yu Chen, Faruque Parvez, Brandon Pierce, Hongyuan Cao, Briseis Aschebrook-Kilfoy, Alauddin Ahmed, Tariqul Islam, Vesna Slavcovich, Mary Gamble, Parvez I. Haris, Joseph H. Graziano, Habibul Ahsan

**Affiliations:** 1 Department of Health Studies, The University of Chicago, Chicago, Illinois, United States of America; 2 Department of Epidemiology, Mailman School of Public Health, Columbia University, New York, New York, United States of America; 3 Department of Environmental Health Sciences, Mailman School of Public Health, Columbia University, New York, New York, United States of America; 4 Department of Environmental Medicine, New York University, New York, New York, United States of America; 5 U-Chicago Research Bangladesh, Ltd., Mohakhali, Dhaka, Bangladesh; 6 Faculty of Health & Life Sciences, De Montfort University, Leicester, United Kingdom; 7 Departments of Medicine and Human Genetics and Comprehensive Cancer Center, The University of Chicago, Chicago, Illinois, United States of America; University of Louisville, United States of America

## Abstract

**Background:**

We utilized data from the Health Effects of Arsenic Longitudinal Study (HEALS) in Araihazar, Bangladesh, to evaluate the association of steamed rice consumption with urinary total arsenic concentration and arsenical skin lesions in the overall study cohort (N=18,470) and in a subset with available urinary arsenic metabolite data (N=4,517).

**Methods:**

General linear models with standardized beta coefficients were used to estimate associations between steamed rice consumption and urinary total arsenic concentration and urinary arsenic metabolites. Logistic regression models were used to estimate prevalence odds ratios (ORs) and their 95% confidence intervals (CIs) for the associations between rice intake and prevalent skin lesions at baseline. Discrete time hazard models were used to estimate discrete time (HRs) ratios and their 95% CIs for the associations between rice intake and incident skin lesions.

**Results:**

Steamed rice consumption was positively associated with creatinine-adjusted urinary total arsenic (β=0.041, 95% CI: 0.032-0.051) and urinary total arsenic with statistical adjustment for creatinine in the model (β=0.043, 95% CI: 0.032-0.053). Additionally, we observed a significant trend in skin lesion prevalence (P-trend=0.007) and a moderate trend in skin lesion incidence (P-trend=0.07) associated with increased intake of steamed rice.

**Conclusions:**

This study suggests that rice intake may be a source of arsenic exposure beyond drinking water.

## Introduction

Rice has been implicated as a dietary source of arsenic (As) exposure [1,2] particularly in South and Southeast Asian countries where rice constitutes the primary source of caloric intake [3]. Rice arsenic concentrations vary widely depending on the rice cultivars and arsenic concentration of water used in irrigation [4]. The speciation (organic versus inorganic) of arsenic in rice varies greatly depending on the variety of rice and on geographical variation [5,6]. In Bangladesh, the primary type of arsenic found in rice is inorganic arsenic (InAs), which is readily absorbed in the blood stream [5,7,8] . Inorganic arsenic is a class I human carcinogen and has been linked to adverse health outcomes including cancers, cardiovascular disease and skin lesions in several exposed populations worldwide, including Bangladesh [9-15]. Inorganic arsenic, once consumed, undergoes metabolic changes into monomethyl arsonate (MMA) and dimethyl arsenate (DMA). The distribution of urinary arsenic metabolites varies from person to person [16,17] and inter-individual variability in the distribution of arsenic metabolites may potentially be impacted by a variety of host, genetic and dietary factors [4,7,18]. Multiple studies have documented presence of inorganic arsenic in rice grains grown, sold and consumed in different parts of the world [6,7,19-21]. 

Two recent small studies investigated associations between rice intake and urinary arsenic in humans [4,22]. The first study, assessing the impact of rice consumption and urinary arsenicals among multiethnic residents in the United Kingdom, found that the median values for urinary As metabolites were higher in UK residents of Bangladeshi origin (which were also reported to have higher rice consumption) as compared to white Caucasians [22]. The second study, conducted in the United States, showed a positive association between rice consumption and total urinary arsenic concentration in a sample of 229 pregnant women [4], indicating that rice consumption should be considered in arsenic reduction strategies in the US and potentially worldwide [4]. However, this study was limited to women with a narrow range of drinking water arsenic concentrations (≤0.07~100 μg/L). 

 Studies of rice consumption in areas such as Bangladesh have shown that rice cultivated using groundwater contaminated by high levels of arsenic often contains relatively higher amounts of arsenic, most of which is found in its more toxic inorganic form [7,18]. Researchers have also modeled the contribution of rice consumption to the dietary intake of arsenic in Bangladesh, where rice is a subsistence food, and have shown that rice is likely to be a major source of dietary arsenic intake for these populations, particularly for populations with low exposure to arsenic via drinking water [5,7,18]. 

The Health Effects of Arsenic Longitudinal Study (HEALS) was initiated in 2000 to prospectively investigate the relationship between chronic arsenic exposure and adverse health outcomes in a population chronically exposed to arsenic via contaminated drinking water. In this study, we evaluated the association of steamed rice consumption, assessed by a validated food frequency questionnaire, with urinary total arsenic in the overall HEALS cohort (N=18,470) and with urinary arsenic metabolites in a subset of the cohort (N= 4,517) from a well-defined geographic region of rural Bangladesh. We also evaluated the impact of steamed rice consumption on arsenic related health outcomes by investigating associations with prevalent and incidence skin lesions in this population. 

## Materials and Methods

### The Health Effects of Arsenic Longitudinal Study (HEALS)

Participants for this study were a part of HEALS, which is a prospective cohort study of a population-based sample of adults in Araihazar, Bangladesh. Detailed information on study methodology and sampling has been previously described [14]. In short, a sampling frame was created by collecting water samples, geographic data, and basic demographic information on the users of 5,966 contiguous wells in a 25 sq-km study area [23]. Between October 2000 and May 2002, 11,746 married individuals aged 18 to 75 were recruited for inclusion in the cohort. Participants underwent standardized interviews administered by trained study physicians that collected information regarding demographic characteristics, diet and well water consumption. Subsequently, an additional 8,287 individuals were recruited during 2006-2008 using the same study methodologies. Study physicians also conducted physical examinations to detect the presence of any arsenic-related skin lesions or symptoms of other related health outcomes. In addition to the interview data, study personnel collected urine and blood samples from participants in their homes, according to a structured protocol [14]. Follow-up for this cohort is ongoing. Physical examinations and structured questionnaires are administered biennially subsequent to enrollment by trained physicians following the same procedures as the baseline examinations. 

### Ethics Statement

 Informed consent procedures have been described previously [12]. Oral consent was obtained from study participants in the presence of a witness for their information to be stored in the University of Chicago and Columbia University hospital database and used for research. Study data is available for research purposes upon request. Consenting procedures were administered orally in their native language and documented by interviewing physicians and a witness. The study and consent procedures were approved by the Ethical Committee of the Bangladesh Medical Research Council and the Institutional Review Boards of Columbia University and the University of Chicago. 

### Dietary Intake of Rice

Dietary intakes of rice were assessed by trained interviewers at baseline using a validated 39-item food frequency questionnaire (FFQ) that was designed for and validated in the HEALS population [24]. Total energy intake was estimated using the US Department of Agriculture (USDA) National Nutrient Database for Standard Reference [25]. Average consumption of each food item in grams per day was calculated for each of the validated FFQ items. The primary source of rice intake for the population was steamed rice. Data for puffed rice and water rice consumption were also available for this population, however results were qualitatively similar to steamed rice, and are therefore not presented here. 

### Measurement of urinary total arsenic and arsenic metabolites

 Among the 20,003 participants, 19,309 participants provided a spot urine sample at baseline. All 19,309 urine samples were analyzed for urinary total arsenic and urinary creatinine, which was used to adjust for urine dilution. A random subset of these individuals were selected for further analysis of urinary arsenic metabolites (N=4,814). After collection, urine samples were stored in coolers until their transfer to -20°C freezers and were batch shipped from the study site on dry ice to Columbia University for analysis. Urinary total arsenic concentration was measured via graphite furnace atomic absorption using the Analyst 600 graphite furnace system (with a detection limit of 2 µg/L). Urinary creatinine was analyzed using a method based on the Jaffe reaction and was used to adjust urinary total arsenic concentration for urine dilution [26]. 

 Urinary arsenic metabolites were measured utilizing a method previously described by Reuter et al [27], which uses high-performance liquid chromatography separation of arsenobetaine, arsenocholine, As^V^, As^III^, MMA and DMA followed by detection by inductively coupled plasma-mass spectrometry with dynamic reaction cell. MMA and DMA exist in two different valence states; however, we did not attempt to distinguish the valence in this study. Additionally, because As^III^ can oxidize to As^V^ during sample transport, storage and preparation, we express As^III^ + As^V^ as total inorganic arsenic (InAs). The percentage of InAs, MMA, and DMA in urine was calculated after subtracting arsenobetaine and arsenocholine from the total [27]. 

### Assessment of water arsenic and other covariates

Well water arsenic concentrations of all of the tube wells assessed in the study were measured using laboratory-based methods previously described [28,29]. Briefly, water arsenic concentrations were measured using graphite furnace atomic absorption spectrometry with a detection limit of 5 µg/L. Samples below the limit of detection were subsequently reanalyzed by inductively coupled plasma mass spectrometry with a detection limit of 0.1 µg/L [28]. At baseline, participants were asked to identify the well used as their primary source of drinking water. Corresponding well water arsenic exposure was subsequently assigned based on this response. 

All covariate data was derived from the baseline interview. Sociodemographic and lifestyle factors included age (continuous years), gender, smoking status (never, current, former), quintiles of well water arsenic concentration, years of education (continuous), skin lesion status (yes/no) and total daily water consumption (mL/day continuous). Body mass index (BMI; weight (kg)/height^2^ (m^2^)) was derived from measured height and weight at baseline. Standard international cutoff points were used to classify participants into underweight (<18.5), healthy weight (18.5-24.9), and overweight (≥25.0) categories. Well water cutoff points for the first and second well water arsenic concentration quintiles at baseline were adjusted to correspond to the WHO’s guideline for arsenic in drinking water (≤10 μg/L) and the national standard for arsenic in drinking water in Bangladesh (≤50 μg/L), respectively. An indicator for study cohort was also included as a covariate in each analysis. 

### Skin lesion assessment

Ascertainment of skin lesions in HEALS is described in detail elsewhere [14]. Briefly, a structured protocol was used to ascertain skin lesions by the study physicians who had undergone training for the detection and diagnosis of skin lesion. The study physicians examined each subject and recorded presence or absence of skin lesions in each body segment at baseline, location of skin lesions, and their size and shape at baseline (prevalent skin lesions) and each biennial follow-up visit (incident skin lesions).

### Exclusions and Eligibility

Among the 20,033 participants overall and the subset of 4,814 with urinary metabolites, only those with available information regarding all outcomes (urinary total arsenic, urinary arsenic metabolites and skin lesions), steamed rice consumption and covariates were included in the analysis. This resulted in 18,470 participants in the overall population and 4,517 in the urinary metabolites subset eligible for these analyses. The overall population included 835 individuals with prevalent skin lesions at baseline and 886 individuals with incident skin lesions across all follow-up visits. 

### Statistical Analyses

The associations between the steamed rice intake and log-transformed arsenic (to approximate a normal distribution) were estimated using generalized linear models with standardized (having a mean of 0 and standard deviation of 1) beta coefficients. Separate models were utilized to evaluate this relationship for creatinine adjusted urinary total arsenic (μg/g), urinary total arsenic (μg/g) (with statistical adjustment for creatinine), DMA%, MMA%, InAs%, MMA (μg/L), DMA(μg/L) , and InAs (μg/L). Initial analyses were sex, age and cohort adjusted. Multivariate models were additionally adjusted for BMI, water arsenic intake, skin lesion status, smoking, total caloric intake and total water consumption per day. Associations between tertiles of steamed rice intake and urinary total arsenic (with and without statistical adjustment for creatinine) were also evaluated using multivariate models. 

Steamed rice values were subsequently utilized to evaluate multiplicative interaction with gender and well water arsenic concentration in relation to urinary total arsenic and metabolites. For the purposes of interaction analyses, well water arsenic concentration was dichotomized into two categories representing less than and greater than the national standard for arsenic in drinking water in Bangladesh (50 μg/L). Multiplicative interaction was implemented by including a cross-product term of the standardized linear rice intake variable and the binary gender or water arsenic variable in the fully adjusted model. The p-value corresponding to this cross-product term was interpreted as the p for interaction. 

We also evaluated the association between steamed rice intake and both prevalent and incident skin lesions in the overall cohort. Logistic regression models were used to estimate prevalence odds ratios and their 95% CIs for the associations between rice intake and prevalent skin lesions at baseline. Discrete time hazard models were used to estimate discrete time hazard ratios and their 95% CIs for the associations between rice intake measured at baseline and incident skin lesions identified at each biennial follow-up examination [30]. Discrete time hazard is defined here as the probability that an individual will develop a skin lesion in each biennial follow-up cycle, conditioned on that individual having been lesion free at the previous study interval. The conditional probability of a new lesion was estimated using a log-linear model with a different intercept for each follow-up wave, but common regression coefficients across all waves. Participants who did not develop skin lesions were censored at the third biennial follow-up or at the time of last skin examination. For the purposes of these analyses, once an individual was censored, there was no re-entry into the analysis cohort. Multivariate models utilized both standardized coefficients and tertiled steamed rice intake (based on the distribution of steamed rice intake at baseline). These models were stratified by a higher baseline well water arsenic concentration (<100 μg/L and ≥ 100 μg/L) due a limited number of reported deaths in individuals with < 50 μg/L. All analyses were conducted using Stata version 11 (Stata Corporation, College Station, Texas). 

## Results

 The distribution of socio-demographic characteristics and rice intake for the overall study population (N=18,470), as well as stratified by urinary arsenic categories, are shown in Table **1**. In the overall population, over 50% of the participants had a well water arsenic exposure of <50 µg/L, and mean well water arsenic concentration increased with increasing categories of urinary arsenic. Among individuals in the lowest category of urinary total arsenic (<37 µg/L) approximately 64% also fell into the lowest category of water arsenic concentration. Similarly, 38% of individuals with the highest urinary total arsenic (> 205 µg/L) fell into the highest category of water arsenic concentration. Distributions of the remaining covariates and rice consumption measures remained relatively consistent across all quintiles of urinary total arsenic. Similar distributions were observed for the urinary metabolites subset (N=4,517) shown in Table **2**. In general, mean water arsenic concentration and age were slightly higher in the urinary metabolites subset compared to the overall population, while steamed rice consumption and years of education were slightly higher in the overall population compared to the subset with metabolite data. 

**Table 1:** Participant characteristics for Overall Population.

		Urinary Total Arsenic (μg/L)*				
**Characteristic**	**Overall**	**Q1 (<37)**	**Q2 (37-65)**	**Q3** (66-113)	**Q4 (114-204)**	**Q5 (205+)**
	**N=18,470**	**N=4838**	**N=3775**	**N=3723**	**N=3230**	**N=2904**
**Water arsenic , μg/L**	**N (%**)	**N (%**)	**N (%**)	**N (%**)	**N (%**)	**N (%**)
Mean (SD)	80.33 (105.15)	26.06 (60.35)	49.22 (74.72)	75.61 (90.52)	109.38 (99.78)	186.44 (132.82)
0.1–10	5724(30.99)	3106(64.2)	1455(38.54)	809(21.73)	279(8.64)	75(2.58)
10.1–50	4254(23.03)	1044(21.58)	1134(30.04)	1077(28.93)	735(22.76)	264(9.09)
50.1–100	3322(17.99)	359(7.42)	648(17.17)	886(23.8)	929(28.76)	500(17.22)
100.1–200	2980(16.13)	218(4.51)	358(9.48)	654(17.57)	789(24.43)	961(33.09)
≥ 200.1	2190(11.86)	111(2.29)	180(4.77)	297(7.98)	498(15.42)	1104(38.02)
**Age, years**						
Mean (SD)	36.92 (10.42)	36.780 (10.30)	37.05 (10.30)	36.89 (10.51)	37.22 (10.54)	36.54 (10.51)
18–30	6216(33.65)	1626(33.61)	1261(33.4)	1249(33.55)	1063(32.91)	1017(35.02)
31–40	5877(31.82)	1553(32.1)	1168(30.94)	1188(31.91)	1021(31.61)	947(32.61)
41–50	3604(19.51)	958(19.8)	790(20.93)	712(19.12)	625(19.35)	519(17.87)
51–75	2773(15.01)	701(14.49)	556(14.73)	574(15.42)	521(16.13)	421(14.5)
**BMI, kg/m^2^**						
Mean (SD)	19.81 (3.19)	19.97 (3.24)	19.92 (3.19)	19.77 (3.23)	19.68 (3.14)	19.56(3.09)
<18.5	7235(39.17)	1772(36.63)	1415(37.48)	1494(40.13)	1320(40.87)	1234(42.49)
18.5-24.9	9906(53.63)	2694(55.68)	2089(55.34)	1956(52.54)	1695(52.48)	1472(50.69)
≥25	1329(7.2)	372(7.69)	271(7.18)	273(7.33)	215(6.66)	198(6.82)
**Education, years**						
Mean(SD)	3.49(3.85)	3.83 (3.98)	3.56 (3.89)	3.40 (3.81)	3.32 (3.83)	3.20 (3.63)
0	7996(43.29)	1944(40.18)	1629(43.15)	1642(44.1)	1453(44.98)	1328(45.73)
1-5	5649(30.58)	1495(30.9)	1121(29.7)	1146(30.78)	988(30.59)	899(30.96)
6+	4825(26.12)	1399(28.92)	1025(27.15)	935(25.11)	789(24.43)	677(23.31)
**Smoking**						
Never	12475(67.54)	3408(70.44)	2518(66.7)	2538(68.17)	2074(64.21)	1937(66.7)
Former	1159(6.28)	259(5.35)	242(6.41)	225(6.04)	225(6.97)	208(7.16)
Current	4836(26.18)	1171(24.2)	1015(26.89)	960(25.79)	931(28.82)	759(26.14)
Steamed rice intake, g/day**						
Mean (SD)	1782.26 (663.43)	1802.62 (654.52)	1831.34 (695.28)	1776.00 (649.32)	1764.20 (671.11)	1752.96(678.38)
0-1550	9701(52.52)	2460(50.85)	1897(50.25)	1986(53.34)	1765(54.64)	1593(54.86)
1551-1950	6535(35.38)	1777(36.73)	1348(35.71)	1315(35.32)	1083(33.53)	1012(34.85)
1951+	2234(12.1)	601(12.42)	530(14.04)	422(11.33)	382(11.83)	299(10.3)

* urinary total arsenic cutpoints were determined by tertiles of study population

** steamed rice consumption cutpoints determined by distribution of consumption in total study population

**Table 2:** Participant characteristics for Metabolites Subset.

		Urinary Total Arsenic (μg/L)*				
**Characteristic**	**Overall**	**Q1 (<37)**	**Q2 (37-65)**	**Q3** (66-113)	**Q4 (114-204)**	**Q5 (205+)**
	**N=4,517**	**N = 913**	**N =831**	**N= 916**	**N=942**	**N=915**
**Water arsenic , μg/L**	**N (%**)	**N (%**)	**N (%**)	**N (%**)	**N (%**)	**N (%**)
Mean (SD)	104.20 (120.41)	37.16 (80.21)	63.97 (90.80 )	91.20 (106.91)	122.17 (105.81)	202.16 (136.72)
0.1–10	1051(23.27)	536(58.71)	264(31.77)	178(19.43)	56(5.94)	17(1.86)
10.1–50	972(21.52)	199(21.8)	249(29.96)	232(25.33)	210(22.29)	82(8.96)
50.1–100	817(18.09)	81(8.87)	158(19.01)	215(23.47)	238(25.27)	125(13.66)
100.1–200	875(19.37)	55(6.02)	95(11.43)	183(19.98)	261(27.71)	281(30.71)
≥ 200.1	802(17.76)	42(4.6)	65(7.82)	108(11.79)	177(18.79)	410(44.81)
**Age, years**						
Mean (SD)	40.81 (10.70)	40.58 (10.41)	40.63 (10.43)	40.88 (10.60)	41.29 (11.01)	40.61 (10.98)
18–30	963(21.32)	195(21.36)	182(21.9)	180(19.65)	201(21.34)	205(22.4)
31–40	1351(29.91)	282(30.89)	242(29.12)	282(30.79)	264(28.03)	281(30.71)
41–50	1046(23.16)	210(23)	196(23.59)	227(24.78)	219(23.25)	194(21.2)
51–75	1157(25.61)	226(24.75)	211(25.39)	227(24.78)	258(27.39)	235(25.68)
**BMI, kg/m^2^**						
Mean (SD)	19.54 (3.24)	19.65 (3.29)	19.69 (3.22)	19.59(3.34)	19.57 (3.26)	19.19(3.02)
<18.5	1967(43.55)	372(40.74)	337(40.55)	407(44.43)	406(43.1)	445(48.63)
18.5-24.9	2264(50.12)	482(52.79)	443(53.31)	434(47.38)	481(51.06)	424(46.34)
≥25	286(6.33)	59(6.46)	51(6.14)	75(8.19)	55(5.84)	46(5.03)
**Education, years**						
Mean(SD)	3.20 (3.80)	3.34 (3.94)	3.36 (3.85)	3.11 (3.75)	3.26 (3.88)	2.93 (3.56)
0	2140(47.38)	427(46.77)	381(45.85)	444(48.47)	436(46.28)	452(49.4)
1-5	1315(29.11)	265(29.03)	242(29.12)	261(28.49)	278(29.51)	269(29.4)
6+	1062(23.51)	221(24.21)	208(25.03)	211(23.03)	228(24.2)	194(21.2)
**Smoking**						
Never	2323(51.43)	493(54)	423(50.9)	479(52.29)	462(49.04)	466(50.93)
Former	463(10.25)	88(9.64)	79(9.51)	87(9.5)	110(11.68)	99(10.82)
Current	1731(38.32)	332(36.36)	329(39.59)	350(38.21)	370(39.28)	350(38.25)
Steamed rice intake, g/day **						
Mean (SD)	1628.17 (620.13)	1656.24 (620.10)	1637.40 (653.90)	1635.51 (583.32)	1609.24 (627.55)	1608.88 (616.74)
0-1550	2826(62.56)	544(59.58)	517(62.21)	566(61.79)	616(65.39)	583(63.72)
1551-1950	1365(30.22)	294(32.2)	248(29.84)	295(32.21)	257(27.28)	271(29.62)
1951+	326(7.22)	75(8.21)	66(7.94)	55(6)	69(7.32)	61(6.67)

* urinary total arsenic cutpoints were determined by quintiles of study population

** steamed rice consumption cutpoints determined by distribution of consumption in total study population

Linear regression analyses evaluating the associations between consumption of steamed rice in relation to urinary total arsenic and urinary metabolites in the overall and subset populations are summarized in Table **3**. Standardized coefficients are reported from each model and represent one standard deviation increase in intake. After multivariate adjustment, steamed rice intake was positively associated with creatinine-adjusted urinary total arsenic μg/g (β=0.041, 95% CI: 0.032-0.051) and urinary total arsenic µg/L with statistical adjustment for urinary creatinine in the model (β=0.043, 95% CI: 0.032-0.053) in the overall population. Similar associations were observed for steamed rice intake in relation to creatinine adjusted urinary total arsenic and urinary total arsenic with statistical adjustment for creatinine in the subset population. Additionally, positive associations were observed for DMA µg/L (β=0.044, 95% CI: 0.008-0.080) and InAs µg/L (β=0.049, 95%CI: 0.013-0.085) in the sex-, age- and cohort- adjusted models, but these associations did not persist after full adjustment in multivariate models. InAs%, MMA% and DMA% were not significantly associated with steamed rice consumption measures and therefore these results are not presented here. Associations between tertiles of steamed rice intake and urinary total arsenic (creatinine adjusted and with statistical adjustment for creatinine) were consistent with the results from the linear models in the overall population (Table **4**). 

**Table 3:** Associations between steamed rice intake and urinary arsenic measures for total population (18,470) and for subset with metabolites data ( N=4,517).

		** **Steamed rice intake (overall population**)					
**Log (outcome)**	**N**	**Sex-, age and cohort-adjusted Beta**	**95% CI**	**P**	**Multivariate adjusted Beta**	**95% CI**	**P**
Cr-adjusted urinary total arsenic (μg/g)	18,470	0.069	(0.055-0.082)	<0.001	0.041	(0.032-0.051)	<0.001
Urinary total arsenic (μg/L) *	18,470	0.068	(0.055-0.083)	<0.001	0.043	(0.032-0.053)	<0.001
		** **Steamed rice intake ( metabolites subset**)					
Cr-adjusted urinary total arsenic (μg/g)	4,517	0.059	(0.031-0.087)	<0.001	0.036	(0.015-0.056)	0.001
Urinary total arsenic (μg/L) *	4,517	0.051	(0.022-0.079)	<0.001	0.031	(0.010-0.052)	0.005
MMA(μg/L)	4,517	0.017	(-0.021-0.054)	0.38	-0.010	(-0.039-0.021)	0.71
DMA(μg/L)	4,517	0.044	(0.008-0.080)	0.02	0.010	(-0.023-0.036)	0.16
Inorganic arsenic (μg/L)	4,517	0.049	(0.013-0.085)	0.01	0.025	(-0.004-0.050)	0.10

Multivariate models adjusted for sex, age, study cohort, BMI, total water intake, skin lesion status, rice consumption(yes/no), water arsenic, education length and smoking status

** Estimates are per standard deviation increase

* Sex- and age-adjusted and multivariate models additionally adjusted for urinary creatinine

**Table 4:** Associations between tertiles of steamed rice intake and urinary arsenic measures for total population.

**(N=18,470**)					
		Steamed Rice intake ( Tertiles)			
		0-1550 g/day	1551-1950 g/day	1951+ g/day	p-trend
Cr-adjusted urinary total arsenic (μg/g)		(Ref)	0.05 (0.02-0.07)	0.11(0.08-0.14)	<0.001
Urinary total arsenic (μg/L) *		(Ref)	0.06 (-0.04-0.1)	0.12 (0.09-0.15)	<0.001

Multivariate models adjusted for sex, age, study cohort, BMI, total water intake, skin lesion status, rice consumption(yes/no), water arsenic, education length and smoking status

* multivariate model also adjusted for urinary creatinine

 Effect modifications of the associations between steamed rice intake and urinary total arsenic and metabolites were evaluated by gender and well water arsenic concentration. No significant interactions for steamed rice consumption with either gender or well water arsenic concentration (dichotomized as <50 µg/L and ≥50 µg/L) were observed in this population (Table **5**). 

**Table 5:** Associations between steamed rice intake and urinary arsenic measures, stratified by sex and water arsenic intake for overall cohort (N=18,470) and subset with urinary arsenic metabolites data (N=4,517).

	** **Steamed rice intake by sex**								
**Log (outcome)**	**Female**				**Male**				
	**N**	**Beta**	**95% CI**	**P**	**N**	**Beta**	**95% CI**	**P**	**P for interaction**
						**Overall Population**			
Cr-adjusted urinary total arsenic (μg/g)	10,926	0.04	(0.03-0.06)	<0.001	7,544	0.04	(0.03-0.05)	<0.001	0.48
Urinary total arsenic (μg/L) *	10,926	0.04	(0.03-0.05)	<0.001	7,544	0.04	(0.03-0.06)	<0.001	0.81
						**Metabolites Subset**			
Cr-adjusted urinary total arsenic (μg/g)	2,038	0.03	(0.01-0.05)	0.01	2479	0.04	(0.02-0.06)	0.001	0.48
Urinary total arsenic (μg/L) *	2,038	0.03	(-0.01-0.05)	0.06	2479	0.03	(0.01-0.60)	0.004	0.33
MMA(μg/L)	2,038	-0.02	(-0.05- 0.01)	0.27	2479	0.001	(-0.03-0.03)	0.96	0.20
DMA(μg/L)	2,038	0.010	(-0.02-0.05)	0.42	2479	0.020	(-0.01-0.06)	0.10	0.37
Inorganic arsenic (μg/L)	2,038	0.003	(-0.02-0.04)	0.84	2479	0.006	(-0.03-0.04)	0.70	0.84
	** **Steamed rice intake by water arsenic**								
**Log (outcome)**	<50 μg/L				≥50 μg/L				
	**N**	**(Beta)**	**95% CI**	**P**	**N**	(**Beta**)	**95% CI**	**P**	**P for interaction**
	**Overall Population**								
Cr-adjusted urinary total arsenic (μg/g)	9,934	0.03	(0.02-0.4)	<0.001	8,536	0.04	(0.02-0.05)	<0.001	0.56
Urinary total arsenic (μg/L) *	9,934	0.02	(0.01-0.03)	<0.001	8,536	0.021	(0.01-0.04)	0.004	0.73
		**Metabolites Subset**							
Cr-adjusted urinary total arsenic (μg/g)	2,007	0.03	(0.01-0.05)	0.01	2510	0.03	(-0.001 - 0.05)	0.06	0.89
Urinary total arsenic (μg/L) *	2,007	0.02	(0.02-0.04)	<0.001	2510	0.02	(0.01-0.04)	<0.001	0.64
MMA(μg/L)	2,007	0.003	(-0.03-0.04)	0.87	2510	-0.001	(-0.04-0.04)	0.97	0.83
DMA(μg/L)	2,007	0.030	(-0.01-0.06)	0.11	2510	0.030	(-0.01-0.07)	0.20	0.49
Inorganic arsenic (μg/L)	2,007	-0.003	(-0.03-0.03)	0.79	2510	-0.002	(-0.03-0.03)	0.79	0.80

Multivariate models adjusted for sex, age, BMI,study cohort, total water intake, skin lesion status, water arsenic, education length, and smoking status

* models additionally adjusted for urinary creatinine

** Estimates are per standard deviation increase in rice intake

The associations for prevalent and incident skin lesions in relation to steamed rice intake are shown in Table **6**. Among individuals with well water arsenic concentration <100 µg/L, a significant association between steamed rice intake and prevalent skin lesions was observed (P-trend=0.007). Similarly, a marginally significant association between steamed rice intake and incident skin lesion was observed among individuals with lower well water arsenic concentration. 

**Table 6:** Associations between steamed rice intake and prevalent and incident skin lesions, stratified by well water arsenic concentration (N=18,740).

		< 100 µg/L		≥ 100 µg/L	
**Prevalent Skin Lesions**		# Skin Lesions	OR (95%CI)	# Skin Lesions	OR(95%CI)
Steamed rice (standardized linear)		424	1.61 (1.17-2.20)	411	1.32 (0.98-1.80)
Steamed rice tertiles					
	0-1550 g/day)	154	1.00	155	1.00
	(1551-1950 g/day)	108	1.33 (1.00-1.76)	121	1.23 (0.92-1.68)
	(1951+ g/day)	162	1.64(1.12-2.40)	135	1.31 (0.87-1.93)
	p-trend		0.007		0.14
**Incident Skin Lesions**		# Skin Lesions	HR(95%CI)	# Skin Lesions	HR(95%CI)
Steamed rice (standardized linear)		446	1.12 (0.90-1.41)	440	0.93 (0.73-1.19)
Steamed rice tertiles					
	(0-1550 g/day)	166	1.00	171	1.00
	(1551-1950 g/day)	122	1.12 (0.87-1.44)	122	0.85 (0.66-1.11)
	(1951+ g/day)	158	1.35 (0.98-1.90)	147	0.86 (0.60-1.21)
	p-trend		0.07		0.35

Adjusted for water arsenic, age, energy intake, education length, BMI, gender, smoking status, and study cohort.

## Discussion

 In this large population-based study, we observed significant associations of steamed rice intake with urinary total arsenic and creatinine-adjusted urinary total arsenic in a Bangladeshi population, for whom rice is the single major staple food. These findings suggest that the population of Bangladesh (and potentially other countries) with known exposure to arsenic through drinking water may be additionally exposed to arsenic through rice consumption, even after taking into account individual arsenic exposure via drinking water.

 A small study by Cascio et al. assessing the impact of rice consumption and urinary arsenicals among UK residents found that the median values for urinary metabolites were higher among residents of Bangladeshi origin as compared to their Caucasian counterparts [22]. This Bangladeshi population was also reported to have higher rice consumption than the white Caucasian population, suggesting that rice consumption contributed to arsenic exposure in this group. The study also showed that the sum of medians of DMA, MMA and InAs for the Bangladeshi group was over 3-fold higher than for the Caucasian group [22]. A recent small US study (n=229) conducted among pregnant women found an association between rice intake and urinary arsenic, suggesting that individuals in the US may be exposed to potentially harmful levels of arsenic exposure through consumption of rice [4]. Arsenic concentrations in drinking water (household tap water) for the pregnant women in this study ranged only from <0.07 µg/L to approximately 100 µg/L [4]. The present study evaluates these associations in over 18,000 individuals with data regarding urinary total arsenic, and over 4,500 individuals with data regarding urinary arsenic metabolites. Rice constitutes the single most important contributor to individual caloric intake in Bangladesh, and this population is ubiquitously exposed to a wide range of arsenic concentrations via drinking water. Therefore, the present study was able to more fully and comprehensively isolate the potential dietary contribution to arsenic exposure. 

The importance of the association between rice consumption and urinary excretion of arsenic metabolites is dependent on the bioavailability (retention, transport and uptake) of arsenic from rice. Inorganic arsenic species are readily disseminated into the blood stream [5,7], and recent studies have shown high bioavailability of inorganic arsenic from cooked rice (>90%) [31]. Studies have investigated the bioaccessibility of arsenicals in rice and measurement of urinary arsenicals within the context of *in vitro* as well as *in vivo* and observational studies [32-35]. 

Urinary total arsenic is a biomarker of individual arsenic exposure [36] and represents the amount of arsenic from all sources (diet, water, soil, etc) that is ingested and excreted. A 2009 study utilizing NHANES data suggests that the U.S. population is not only exposed to arsenic through the consumption of various foods, including fruit juices, vegetables and rice, but that the US population is more exposed to arsenic via food sources than from drinking water, due to the relatively low arsenic concentrations of the drinking water supply [35]. The results of the present study suggest that even after taking into account exposure to arsenic via drinking water, which is high in the population in question, steamed rice intake is significantly associated with urinary total arsenic after adjustment for creatinine, suggesting that rice intake significantly contributes to individual arsenic exposure in this population. These results are consistent with prior studies that suggest the presence and species of arsenic in cooked rice [20,21]. 

The results of the present study also have important implications regarding the health outcomes of this Bangladeshi population. Analysis investigating the association between rice consumption and skin lesions suggests that arsenic concentration in rice plays a key role in overall arsenic exposure, particularly for individuals with low arsenic exposure from drinking water. For these individuals, a greater proportion of their overall arsenic exposure is derived from rice intake. While high doses of arsenic via drinking water may overshadow the potential impact of arsenic exposure through rice, individuals with low baseline exposure may be put at increased risk for adverse health outcomes due to arsenic exposure from rice. 

Evidence for this association was stronger in the case-control analysis of skin lesion prevalence as compared to the discrete time hazards models of skin lesion incidence. The attenuated effect for prospective analysis of skin lesion incidence can potentially be attributed to reduced power or misclassification for detecting incident skin lesions. Incident skin lesions detected during follow-up were likely to be less severe than prevalent skin lesions identified at baseline. Therefore, if some of the milder, incident lesions were not detected as incident skin lesions, then misclassification in this instance may have led to an attenuated association between rice consumption and incident skin lesions. It is also potentially due to the long-term beneficial effect of the nutrients in rice on skin lesion risk. A previous study has suggested that dietary intake of methionine, cysteine and protein resulted in increased urinary excretion of arsenic, taking into account arsenic exposure via drinking water, presumably through improved arsenic methylation [37]. While this cannot be evaluated within the scope of the present analysis, several smaller scale studies have suggested that increased levels of arsenic excretion may not necessarily equate to better metabolism of arsenic [4,22,38]. The impact of individual nutrient intake on arsenic methylation and excretion is an important area for future evaluation. 

In addition to the large sample size and wide dose range of the study population mentioned above, this study has several strengths. First, nearly 100% of this population regularly consumed steamed rice. This, coupled with a virtually ubiquitous exposure to arsenic via drinking water makes it possible to isolate the contribution of arsenic from rice. Secondly, this study population has also been systematically evaluated for arsenic related health outcomes, primarily skin lesions, allowing for the evaluation of the potential impact of rice consumption on these outcomes. Finally, this study utilized a validated FFQ. This study instrument contains the food items most commonly consumed by this Bangladeshi population, and therefore, information regarding rice intake was readily available. 

While the study has several strengths, there are also notable limitations. Despite available data regarding the intake of other food items that could act as possible sources of arsenic exposure, such as fish and vegetables, this paper did not include assessment of associations between the intake of these food items and urinary total arsenic or the excretion of arsenic metabolites. The population utilized in this study is a largely rural and resource poor population with highly variable and seasonal nutritional intake. Their intake of animal proteins and vegetables are generally low. Additionally, there have been extensive studies suggesting high bioavailability of inorganic arsenic in rice [2,8,31] which is considered to be more toxic than the organic form [18]. Elevated levels of arsenic have been detected in fish and seafood, however several studies have reported these to be of the less toxic, organic, species [39-41] . While there is evidence to suggest the bioavailability of inorganic arsenic in plant tissues, this association is less well characterized as are methodologies for arsenic speciation in plant tissues [42]. Understanding potential arsenic exposure through other food items however, is an important area of future research.

 The measurement of arsenic exposure in this population is also limited. Arsenic exposure via rice can potentially be due to water utilized during food preparation and cooking for which we do not have accurate information. Secondary sources of water arsenic exposure outside the primary well were also not considered in this analysis. Arsenic measurements from rice were not available in the scope of the present study. Additionally, we were not able to provide quantitative estimates of the exposure and the variation in exposure to arsenic via rice by geographic regions, type or source of rice, or methods of cooking. Another limitation of this study is that FFQ data measures average diet and does not accurately measure the actual nutritional intake of the individuals. Despite this limitation however, FFQ data is successful in ranking study participants relative to each other regarding exposure. Finally, it is possible that there are discrepancies between intake at the time of interview and urine sample collection and the reported average dietary intake, due to factors such as seasonal variability. 

 The health effects of arsenic exposure through drinking water are well characterized; however, the health effects of arsenic exposure through food sources are much less well-understood. Understanding the amount of arsenic that this population is exposed to via food is critical in controlling adverse health outcomes related to arsenic toxicity in this population and other populations worldwide. Future research is necessary to understand the impact of arsenic exposure from food on specific health outcomes.
